# Effects of Mould Temperature on Rice Bran-Based Bioplastics Obtained by Injection Moulding

**DOI:** 10.3390/polym13030398

**Published:** 2021-01-27

**Authors:** María Alonso-González, Manuel Felix, Antonio Guerrero, Alberto Romero

**Affiliations:** 1 Departamento de Ingeniería Química, Facultad de Química, Universidad de Sevilla, 41012 Sevilla, Spain; alromero@us.es; 2 Departamento de Ingeniería Química, Escuela Politécnica Superior, Universidad de Sevilla, 41011 Sevilla, Spain; mfelix@us.es (M.F.); aguerrero@us.es (A.G.)

**Keywords:** bioplastics, rice bran, injection moulding, water absorption capacity

## Abstract

The high production rate of conventional plastics and their low degradability result in severe environmental problems, such as plastic accumulation and some other related consequences. One alternative to these materials is the production of oil-free bioplastics, based on wastes from the agro-food industry, which are biodegradable. Not only is rice bran an abundant and non-expensive waste, but it is also attractive due to its high protein and starch content, which can be used as macromolecules for bioplastic production. The objective of this work was to develop rice-bran-based bioplastics by injection moulding. For this purpose, this raw material was mixed with a plasticizer (glycerol), analysing the effect of three mould temperatures (100, 130 and 150 °C) on the mechanical and microstructural properties and water absorption capacity of the final matrices. The obtained results show that rice bran is a suitable raw material for the development of bioplastics whose properties are strongly influenced by the processing conditions. Thus, higher temperatures produce stiffer and more resistant materials (Young’s modulus improves from 12 ± 7 MPa to 23 ± 6 and 33 ± 6 MPa when the temperature increases from 100 to 130 and 150 °C, respectively); however, these materials are highly compact and, consequently, their water absorption capacity diminishes. On the other hand, although lower mould temperatures lead to materials with lower mechanical properties, they exhibit a less compact structure, resulting in enhanced water absorption capacity.

## 1. Introduction

Nowadays, research and development of biopolymers has grown due to the increasing interest in using renewable and natural sources in the polymer processing industry. This is a consequence of the depletion of oil reserves and the serious environmental issues caused by plastic accumulation. Biopolymers can be divided into two categories: biodegradable polyesters, which are petroleum-based yet biodegradable, and polymers from renewable sources, such as protein and starch-based biopolymers, which are produced from natural sources and are readily biodegradable [1]. Proteins and starches are widely found in wastes and byproducts from the food and agricultural industries [2,3]. In the best scenario, these materials are used for animal feeding, as they are a low-added-value byproduct, even though this is a better end-of-life option than incineration or composting, which in turn are better than landfill disposal or leakage, which is by far the worst-case scenario. Therefore, biodegradable polymers can make significant contributions to material recovery (producing high added value products), reduction of landfill and utilization of renewable resources [4].

Starch-based biopolymers have gained attention and importance since they show thermoplastic-like processability after the application of suitable temperature and shear. In fact, a well-known type of starch-based polymer is the so-called thermoplastic starch (TPS) [5]. TPS is similar to other synthetic polymers (in terms of structure, molar mass, glass transition temperature, crystallinity, melting temperature, etc.). However, it melts and fluidizes in the presence of a low molecular plasticizer such as water or glycerol, high temperatures and shearing, being suitable for injection moulding, extrusion and blowing facilities, in the same way as synthetic plastics [6]. Similar to starches, proteins are macromolecules with continuous and cohesive matrices, receiving enormous attention for the production of biodegradable plastic, edible films and sheets [7]. Plant proteins that can be used for bio-based plastic include soy protein, corn zein, wheat protein, etc. [7,8,9]. Animal proteins such as blood meal, gelatin, collagen, keratin, egg protein, … etc., can also be used as feedstocks of such bio-based plastics [10,11,12]. For this purpose, plasticizers are generally added to the protein matrix during thermoplastic processes such as extrusion and injection moulding to improve its processability, reduce brittleness and modify the properties of the final structure [13]. Typically, the manufacture of protein-based bioplastics involves chemically, thermally, or pressure-induced protein denaturation as a first stage. Due to the diversity in the assembling of protein networks and their unique structures, a large variety of biodegradable materials can be produced, offering a wide range of functional properties [14]. Although the thermoplastic processing of starch and protein-based plastics is being widely studied, they still have weaknesses in their characteristics, such as weak mechanical properties, poor long-term stability and sensitivity to water; thus, there is scientific interest for studying new bio-based materials and optimizing processing conditions to find well-established alternatives to conventional plastics [15].

In this way, rice bran (RB) is a by-product of brown rice production. It contains up to 20% protein, 45% carbohydrates (mainly starch), 10% fibre and some varying percentages of moisture, lipids and ashes [16]. Globally, rice cultivation covers 145 million ha. In the European Union alone, the area dedicated to this crop is approximately 410,000 ha. Furthermore, it is estimated that 100 million tons of rice residues and byproducts are generated each year [17]. Currently, rice residues are treated as waste products, so they are incinerated for energy purposes or used as animal feed. For these reasons, the composition of rice bran suggests its possible successful employment for bioplastic production, which would benefit from reliable, renewable sources, creating high-value-added products that are also biodegradable, i.e., beneficial for the environment. Bioplastics have already been obtained from rice husk protein concentrate [17]. However, more research is required to produce competitive bio-based plastics from food industry by-products such as RB.

This research work aims to develop RB-based bioplastics via injection moulding, using water and glycerol as plasticizers. Furthermore, the effects of mould temperature on the properties of the final bioplastics were also studied. To this end, rheological measurements and mechanical, functional and microstructural characterization were carried out on the final samples. 

## 2. Materials and Methods

### 2.1. Materials

RB was provided by Herba Ingredients (San José de la Rinconada, Seville, Spain). The RB supplied by this company is a byproduct obtained from the polishing process that produces the variety “vaporized indica white rice”. Water (w) and glycerin (gly) were employed as plasticizers; the former was deionized-grade water, whereas the latter was supplied by PANREAC S.A. (Seville, Spain). All other reagents were supplied by Sigma Aldrich (St. Louis, MO, USA).

### 2.2. Chemical Composition

The chemical composition of RB was characterized by following the A.O.A.C. methods [18]. The protein content was determined as % N × 6.25 using a LECO TRUSPEC CHNS-932 nitrogen micro analyser (Leco Corporation, Saint Joseph, MI, USA) [19]. Ash content was determined by heating a small amount (5 g) of RB at 550 °C in a muffle furnace (Hobersal HD-230, Barcelona, Spain) for 5 h in air atmosphere. The sample was then cooled to room temperature in a desiccator before being weighted again to calculate the mass difference. The lipid content was quantified by the Soxhlet extraction method [20]. For this purpose, hexane was used as the solvent in a Soxhlet device. The hexane in contact with the sample dragged the lipids in subsequent cycles until the whole lipid content was removed. The exact lipid amount was also calculated by mass difference. Moisture was determined by placing 3 g of sample in a conventional oven (Memmert B216.1126, Schwabach, Germany) at 105 °C for 24 h. 

### 2.3. Sample Preparation

Blends containing sieved rice bran (<500 μm), water and glycerin (RB/w/gly) were prepared by a two-stage thermomechanical procedure. Water was used as a plasticizer for starch, since it can break hydrogen bonds, allowing starch gelatinization. However, it cannot be used alone, as the products obtained are brittle, and consequently, it should be used with some other plasticizer, such as glycerol or sorbitol [21]. For this reason, selected blends containing 55% RB and 45% total plasticizer (2:1 w/Gly) were mixed in a two-blade counter-rotating batch mixer HAAKE POLYLAB QC (Thermo Scientific, Waltham, MA, USA) at 200 rpm and 80 °C, which was also selected as the intermediate temperature used by other authors (the process is usually carried out between 70 and 90 °C) [22]. This stage was carried out during 1 h based on a previous research work that employed a variety of rice bran plasticized by extrusion [16]. Secondly, the obtained doughs were processed by injection moulding using a MiniJet Piston Moulding System II (Thermo Scientific, Waltham, MA, USA). The temperature of the cylinder was fixed to 50 °C, and the pressure applied to force the material into the mould cavity was 500 bar; both conditions were applied for 15 s injection time. Three mould temperatures (100, 130 and 150 °C) were employed to study the influence of this parameter on the final properties of the bioplastics. These temperatures were selected based on previous works, and the results obtained from the temperature sweep tests performed on the processed doughs [23,24]. Thus, three systems were obtained, all of them processed at 500 bar injection pressure for 200 s. The fixed processing conditions were selected based on previous works carried out with similar samples. Rectangular bioplastic samples measuring 60 × 10 × 1 mm were obtained for further mechanical and microstructural characterization.

### 2.4. Ageing of Doughs

The obtained doughs were not suitable for injection moulding right after the mixing stage; however, when kept in opened containers with <45% humidity, they evolved, acquiring a firmer appearance to the naked eye and losing some of their moisture content. In this way, after a certain amount of time, they were successfully processed by injection moulding. In order to quantify this ageing process, the homogeneous blends were characterized every 24 h through rheological measurements, while the water content was determined until a steady state was reached. A Dynamic Mechanical Analyser (RSA3, TA Instruments, New Castle, DE, USA) was employed in compression mode using a plate–plate geometry (8 mm diameter). Strain sweep tests were performed in order to establish the linear viscoelastic range. Subsequently, the lower critical strain was selected to carry out frequency sweep tests between 0.01 and 20 Hz at room temperature. Finally, once the dough remained unchanged, a temperature sweep test between 30 and 160 °C at 5 °C/min was also carried out to study the rheological behaviour of the dough to be injected with temperature. In these measurements, the storage modulus (E′), loss modulus (E″) and loss tangent (tan δ) were determined for the whole studied ranges. The water content was characterized following the same protocol mentioned in Section 2.2 for moisture content. 

### 2.5. Bioplastics Characterization

#### 2.5.1. Dynamic Mechanical Thermal Analysis (DMTA)

These tests were performed with an RSA3 dynamic mechanical analyser (TA Instruments, New Castle, DE, USA) on the obtained bioplastics using two grips, one at the bottom and one at the top, to study their rheological behaviour in tension mode. Again, the linear viscoelastic range was first determined, and the lower critical strain was used in the subsequent experiments. Frequency sweep tests at room temperature were performed between 0.01 and 20 Hz for the three manufactured systems (100, 130 and 150 °C mould temperature). Finally, temperature sweep tests at 1 Hz between 30 and 140 °C were carried out for all systems using a heating rate of 5 °C/min. 

#### 2.5.2. Tensile Tests

Tensile tests were performed using the RSA3 device in continuous deformation mode following a modified protocol (using rectangular specimens) based on ISO 570-2:2012, regarding the assessment of mechanical properties of polymeric samples [25]. At least three strain–stress curves were obtained for each evaluated system (100, 130 and 150 °C) and the Young’s modulus (E), maximum tensile strength (σ_max_) and elongation at break (ε_max_) were evaluated with a strain rate of 1 mm/min at room temperature (25 ± 1 °C). 

#### 2.5.3. Water Uptake Capacity and Soluble Matter Loss

Water uptake capacity was measured according to the ASTM D570 method [26], using a third of the rectangular bioplastic specimens, that is, a rectangular sample measuring 20 × 10 × 1 mm. The specimens were dried in a conventional oven (UN 55, Memmert, Schwabach, Germany) at 50 °C for 24 h to determine the dry weight. Immediately after, they were immersed in distilled water for 24 h. Finally, they were submitted to a freeze-drying step at −80 °C under vacuum in a LyoQuest freeze-dryer with a Flask M8 head (Telstar, Seville, Spain) and their weights after these last two steps were employed to determine their water uptake capacity and soluble matter loss according to the following equations:

Water Uptake Capacity (%) = 100·(w_2 −_ w_3_)/w_3_, (1)

Soluble Matter Loss (%) = 100·(w_1_ − w_3_)/w_1_, (2)

where w_1_, w_2_ and w_3_ are the weight of the bioplastic after dehydrothermal treatment, the immersion step and the freeze-drying stage, respectively.

#### 2.5.4. Scanning Electron Microscopy (SEM)

The microstructure of the final bioplastics after the freeze-drying step was studied by scanning electron microscopy using a Zeiss EVO device (Carl Zeiss Microscopy, White Plains, NY, USA) in order to establish a relationship between the processing conditions and the structure of the final specimens. The samples were first coated with Pd/Au (10 nm) by sputtering using a Leica AC600 Metallizer, and then they were observed at 10 kV acceleration voltage and 500× magnification. Finally, the mean crack diameter was calculated using the software ImageJ using at least 5 measurements. 

### 2.6. Statistical Analyses

At least three replicates of each measurement were carried out. Statistical analyses were performed using t-test and one-way analysis of variance (ANOVA) (significance value (*p*) < 0.05) using the STATGRAPHICS 18 software (Statgraphics Technologies, Inc., The Plains, VA, USA). Standard deviations from some selected parameters were calculated. Significant differences are indicated by different letters; that is, all mean values labelled with the same letter did not show significant differences. 

## 3. Results

### 3.1. Chemical Composition

The RB variety analysed (vaporized Indica), shows a composition of 7.06 ± 0.09% moisture, 10.50 ± 0.16% ashes, 13.22 ± 0.52% proteins and 22.77 ± 1.33% lipids. There is a remaining percentage, accounting for 46.45% of the sample, which can be attributed to carbohydrates.

### 3.2. Doughs Characterization (Ageing)

**Figure 1:**
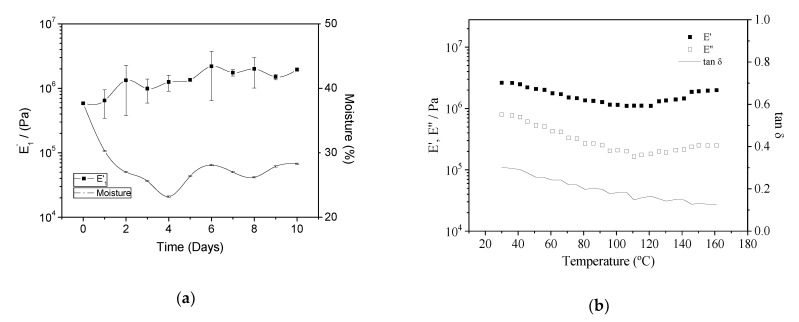
Viscoelastic properties at a constant frequency (1 Hz) of the doughs obtained after mixing. (**a**) Evolution of the elastic modulus (E′) and moisture content (%) over time. (**b**) Temperature sweep test of the stabilized dough between 30 and 160 °C.

Figure 1a shows the ageing process of the doughs obtained after mixing. Two parameters are considered: the elastic modulus (E′) and moisture content (%). E_1′_ are the elastic modulus values at 1 Hz obtained from different frequency sweep tests performed each day for the corresponding doughs. As can be seen, E′ increased with time, reaching its maximum value after 6 days ageing time, with no further increases for longer times. Moreover, the water content followed the same tendency, stabilizing its value after 6 days. However, its values decreased with time from almost 40% moisture right after mixing to less than 30% at the end of the characterization (10 days ageing). 

Once the doughs were stabilized (after 6 days) and their parameters remained constant, they were subjected to temperature sweep tests between 30 and 160 °C. Figure 1b shows the elastic (E′) and viscous (E″) moduli with increasing temperature. Both viscoelastic moduli followed the same behaviour, i.e., a decrease with temperature up to 100–120 °C (E′ decreased from 2.63 × 10^6^ to 1.10 × 10^6^ Pa) and, subsequently, an increase for temperatures above 120 °C (E′ up to 1.99 × 10^6^ Pa), indicating the thermosetting potential of the doughs before their processing by injection moulding [12]. The loss tangent (tan δ) decreased for the whole studied range; thus, no phase transitions were recorded [27]. 

### 3.3. Bioplastics Characterization

#### 3.3.1. Dynamic Mechanical Thermal Analysis (DMTA)

**Figure 2:**
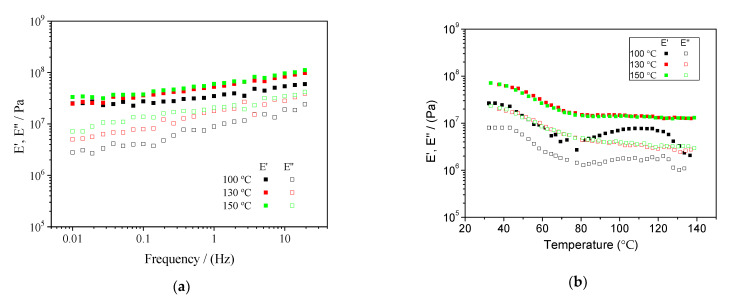
DMTA tests performed on the final bioplastics. (**a**) Frequency sweep tests from 0.01 to 20 Hz at room temperature and (**b**) temperature sweep tests from 30 to 140 °C at 1 Hz.

Figure 2a shows the results of the frequency sweep tests carried out on the systems obtained with different mould temperatures (100, 130 and 150 °C) between 0.01 and 20 Hz. As can be seen, E′ was always higher than E″, indicating the predominantly elastic character of the obtained bioplastics, where both moduli increased with frequency, showing a noticeable dependence. The systems were also subjected to temperature sweep tests between 30 and 140 °C (Figure 2b). Two different behaviours were observed in this figure. First, the systems processed at 130 and 150 °C showed a similar response, with E′ and E″ decreasing with increasing temperature up to 80 °C. From this temperature to 140 °C, none of the two moduli changed significantly (*p* < 0.05). On the other hand, the system processed at 100 °C, although with significantly lower E′ ad E″ values, exhibited a similar behaviour up to 80 °C, with both moduli decreasing with increasing temperature. However, from 80 °C to 110 °C, E′ and E″ increased to values closer to those obtained for the other evaluated systems, being the only samples with certain thermosetting potential [28]. Finally, both viscoelastic moduli exhibited an abrupt decrease for the higher temperatures considered. 

#### 3.3.2. Tensile Tests

**Figure 3:**
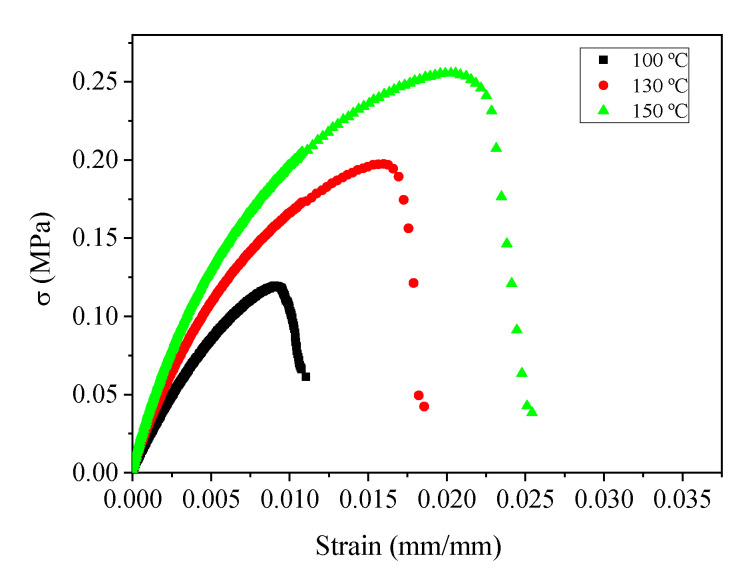
Stress–strain curves obtained from tensile tests for the bioplastics obtained for different mould temperatures (100, 130 and 150 °C).

The stress–strain curves obtained from tensile tests are shown in Figure 3. The mechanical properties of the processed bioplastics improved for higher injection moulding temperatures, increasing the maximum tensile strength and strain at break. Thus, the bioplastics obtained at 130 and 150 °C are stiffer and exhibit greater toughness, especially the system obtained at 150 °C. For a more accurate comparison, Young’s modulus (E), tensile strength (σ_max_) and strain at break (ε_max_) are shown in Table 1 for all studied systems. 

Table 1 shows that all parameters are improved when higher processing temperatures are used. Young’s modulus increased from 12 ± 7 to 23 ± 6 MPa when the temperature increased from 100 to 130 °C, reaching 33 ± 6 MPa for the higher temperature employed (150 °C). Tensile strength also increased from the 0.08 ± 0.04 MPa, exhibited by the sample obtained at 100 °C to 0.27 ± 0.01 MPa for the 150 °C sample, with the 130 °C sample having an intermediate value of 0.18 ± 0.03 MPa. Finally, the elongation at break also improved with temperature, being 1.1 ± 0.3% for the 100 °C sample, 1.6 ± 0.4% for the 130 °C sample and 2.4 ± 0.3% for the sample processed at 150 °C. It is worth mentioning that the elongations exhibited by the samples led to no significant changes in their cross-sections, being below 1% in all cases. Similar results were found by Felix et al. [28] for crayfish-based bioplastics. However, these results are not usually found in protein-based systems, where the Young’s modulus also increases with temperature but the elongation at break decreases [29]. 

#### 3.3.3. Water Uptake Capacity and Soluble Matter Loss

**Figure 4:**
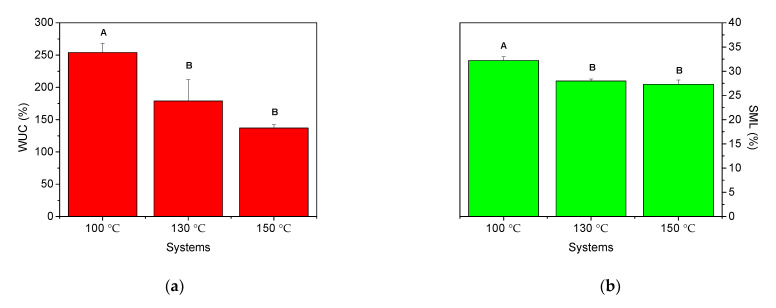
(**a**) Water uptake capacity (WUC) and (**b**) Soluble Matter Loss (SML) for the bioplastics obtained for different mould temperatures (100, 130 and 150 °C). Different letters above each bar indicate significant differences (*p* < 0.05).

The results obtained from the water absorption tests (Figure 4) show that an increase in the injection moulding temperature leads to poorer water uptake capacities. In this way, the water uptake capacity of the systems obtained at 100 °C was 254 ± 14%, while for the systems processed at 130 and 150 °C, these values decreased to 179 ± 33 and 137 ± 5%, respectively. Soluble matter loss also decreased with increasing temperature, although the differences were slighter, changing from 32.2 ± 0.8 to 28.0 ± 0.4 and 27.3 ± 0.9% for the systems obtained at 100, 130 and 150 °C, respectively. 

#### 3.3.4. Scanning Electron Microscopy (SEM)

**Figure 5:**
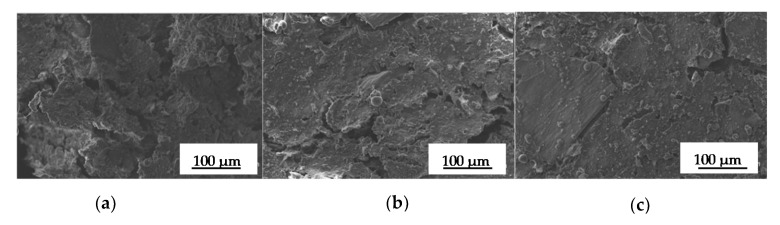
SEM micrographs after lyophilization of the different samples at (**a**) 100 °C; (**b**) 130 °C; (**c**) 150 °C.

Figure 5 shows SEM micrographs after 24 h of water immersion and subsequent freeze-drying of the obtained bioplastics. The SEM images indicate that a continuous structure was obtained, where the homogeneity of the system depends on the processing temperature. Thus, this procedure did not lead to porous matrices but compact bioplastics, where the effect of increasing processing temperature could be analysed. The higher the injection moulding temperature, the more compact the morphology of the bioplastic is, showing a more uniform surface with fewer cracks and with the mean crack diameter decreasing from 121 ± 24 μm for the 100 °C system to 54 ± 16 and 29 ± 16 μm for the 130 and 150 °C systems. The effect was most noticeable when the highest temperature was applied. 

## 4. Discussion

### 4.1. Chemical Composition

The chemical composition of the sample was successfully evaluated, leading to similar results to those obtained by Siswanti et al. [30] and Thiranan et al. [31] for red and defatted rice bran. According to these research works, the majority of the sample accounts for carbohydrates; thus, the non-identified percentage of the rice bran under evaluation can be attributed to this basic food group. Furthermore, the characterization carried out by Klanwan et al. [16] revealed a variety of this byproduct with a very similar composition, which was employed for the same purpose (bioplastic production), suggesting the suitability of rice bran to produce bioplastics obtained by injection moulding. Besides, these authors highlighted the presence of fibre and starch in the carbohydrate fraction, as two of the main components, along with protein, for this valorisation process. This fact supports the importance of using strategies based on these biopolymers for the development of bioplastics.

### 4.2. Doughs Characterization (Ageing)

Under the selected conditions, the obtained doughs achieved a steady state after ageing for six days, and after this time, they were suitable for injection moulding. As can be seen in Figure 1a, the elastic modulus increased when the moisture content decreased; thus, it can be assumed that both parameters are related. In order to determine whether there is some structuration of the doughs during ageing when no water is lost, the doughs were kept in closed containers (the moisture content remained unchanged) and their rheological parameters were measured with time. In this experiment, the obtained doughs did not change, with the elastic modulus remaining constant for one week (results shown in Supplementary Material, Figure S1). Therefore, the moisture content was selected as the main parameter to be considered when the doughs are evaluated before injection moulding. Thus, excess water leads to incomplete filling of the mould cavity, since the water evaporates in the mould, causing voids and cracks. However, water is required during dough formation since it promotes the breakage of the hydrogen bonds, leading to the subsequent drop in glass transition and melting temperatures, improving processability [13].

Regarding the temperature sweep tests shown in Figure 1b, the thermosetting potential exhibited above 120 °C indicates that the mechanical properties of the final bioplastics will probably improve when processing at these higher temperatures [32]. This response can be attributed to the protein fraction that undergoes some heat-induced strengthening phenomena (e.g., aggregation and gelation). In this way, the best results are expected for the systems obtained at 130 and 150 °C, especially for the latter. Further characterization of obtained bioplastics by injection moulding will elucidate this thermomechanical response.

### 4.3. Bioplastics Characterization 

#### 4.3.1. Dynamic Mechanical Thermal Analysis (DMTA)

The frequency sweep tests obtained for the final bioplastics indicate a predominantly elastic character with certain frequency dependence, which has been obtained in previous studies for different successful protein-based biodegradable bioplastics [7,33]. Moreover, the rheological parameters (viscoelastic moduli) improved for higher temperatures, as expected from the results obtained in Section 3.2 regarding the rheological behaviour of the doughs with temperature.

On the other hand, the only system that followed a different tendency when evaluating the rheological properties with temperature was the one processed at 100 °C, whose moduli began to diminish with temperature until an important increase was observed between 80 and 120 °C, indicating the remaining thermosetting potential. It seems very clear that better rheological properties were obtained for higher processing temperatures, with no significant differences between the systems processed at 130 and 150 °C, which followed the same behaviour with also very similar values. In this case, the increase in processing temperature, with the corresponding higher economic and energetic costs, would not be justified, since no further improvements in the rheological parameters were obtained. Similar results for temperature sweep tests were obtained by Felix et al. [23] for bioplastics obtained from albumen, soy and pea protein isolate, as well as rice husk protein concentrate. 

#### 4.3.2. Tensile Tests

The results obtained from the tensile tests shown in Figure 3 and Table 1 confirm the above-mentioned behaviours, where higher processing temperatures produced bioplastics with improved mechanical properties. In this case, the three studied parameters (i.e., σ, ε and E) improved not only when the temperature increased from 100 to 130 °C, but also for the system obtained at 150 °C, which justifies the further increase in temperature that led to better mechanical properties in tension, besides the fact that these differences were not observed in the previous section. Finally, it is worth mentioning that similar Young’s modulus values, although with slightly higher tensile strengths, have been obtained in two previous studies for crayfish, albumen and rice husk materials [23,28], where the authors stated that the protein-based bioplastics exhibited suitable characteristics to replace conventional plastics in different applications. However, the samples obtained in the present research work exhibited higher elongation at break. In addition, the obtained results are very similar to those obtained by Klanwan et al. [16] and Zárate-Ramírez et al. [24] for protein/carbohydrate-based bioplastics, in the first case obtained by extrusion using kraft lignin for plastification purposes, especially to those obtained for the higher kraft lignin content. This research shows the synergetic effect of the combination of proteins and carbohydrates in the biopolymer matrix since the increase in the Young’s modulus also brings an increase in the elongation at break, which is not typically observed in protein-based materials [34]. In any case, all the bioplastics obtained at different temperatures exhibit tensile properties lower than synthetic polymers such as LDPE. Thus, the tensile strength, elongation at break and Young’s modulus show values that reach as much as 2%, 1% and 10%, respectively, of the values for ASTM-normalized LDPE [35]. 

#### 4.3.3. Water Uptake Capacity and Soluble Matter Loss

The water uptake capacity values decreased from 254 to 137% when temperature increased from 100 to 150 °C, indicating that the mechanical properties improved with higher processing temperatures. Thus, the systems with stronger structuration and better mechanical behaviour retain less water within their structure [36]. In this way, the systems with higher physical integrity, that is, those with stronger structure during processing, will undergo minor matter loss during immersion. As can be seen, this can be observed in Figure 4b, which shows the lower soluble matter loss values exhibited for higher temperatures. Finally, it should be highlighted that this behaviour has been observed before in previous studies, where higher mechanical properties led to poorer water uptake capacities and lower soluble matter loss results in pea protein-based bioplastic systems [37]. 

There is a remarkable difference with the results found for bioplastic matrices containing protein as the only biopolymer, which generally release the whole plasticizer content at the immersion stage [29]. These results suggest that the starch fraction contributes to retaining an important proportion of the plasticizer (either water, glycerol or both). 

#### 4.3.4. Scanning Electron Microscopy (SEM)

The micrographs shown in Figure 5 match all results obtained before. Firstly, the most homogeneous structures correspond to those systems that exhibited the highest mechanical properties, which then retained less water and underwent minor matter losses during immersion. Thus, the water uptake capacities and mechanical properties exhibited can be related to the microstructure generated, with greater homogeneity producing higher mechanical properties and lower water absorption. In this sense, the systems with poorer mechanical behaviour and better water absorption properties correspond to the less compact structures, showing more voids and cracks. From these results, it can be assumed that higher processing temperatures might induce more protein–protein interactions, producing more compact structures with higher physical integrity, resulting in improved mechanical properties, as was observed before for wheat gluten-based bioplastics obtained using a very similar process [38]. 

## 5. Conclusions

The results obtained in this work support the suitability of RB to develop bioplastics obtained by injection moulding. The initial high content of carbohydrates (~46%) indicates that the processing conditions selected must be compatible with both proteins and polysaccharides. Consequently, the formulation included not only glycerol as the plasticizer but also water during the formation of doughs, which allows the breakage of hydrogen bonds, increasing processability and allowing thermo-mechanical methods to be employed in the manufacture of the desired bioplastics. Unfortunately, this water content hinders the processing of samples by injection moulding, and thus the dough-like blends were subjected to ageing, obtaining constant and suitable values after 6 days. Once the bioplastics were successfully processed by this technique, the effect of mould temperature was analysed. The obtained results indicate that higher temperatures involved enhanced mechanical properties, which was evidenced by an increase in the elastic moduli (from 3.49 × 10^7^ to 6.01 × 10^7^ Pa for the systems processed at 100 and 150 °C, respectively), as well as an increase in the tensile strength parameters (σ_max_ increased from 0.12 ± 0.04 to 0.26 ± 0.01 MPa for the systems processed at 100 and 150 °C, respectively), which were caused by the development of more protein–protein interactions for higher temperatures. Unfortunately, the increase in the viscoelastic moduli resulted in lower water absorption capacities. In this sense, the greater structuration of the systems coincided with the lower soluble matter loss observed. Lastly, the obtained micrographs confirm the more homogeneous (compact) structure obtained as the temperature increased up to 150 °C, where voids and cracks were absent. This microstructure matches the higher mechanical properties and lower water absorption previously observed, since the continuous structure hindered the water penetration and facilitated the matrix breakdown.

These results confirm the suitability of RB as raw material for the generation of natural bioplastics; however, further research is needed to generate bioplastics for commercial applications.

## Figures and Tables

**Table 1:** Young’s modulus, maximum tensile strength and elongation at break of the different processed systems. Different superscript letters within a column indicate significant differences (*p* < 0.05).

System	Young’s Modulus (MPa)	Maximum Tensile Strength (MPa)	Elongation at Break (%)
100 °C	12 ± 7 ^a^	0.12 ± 0.04 ^a^	1.1 ± 0.3 ^a^
130 °C	23 ± 6 ^a,b^	0.18 ± 0.03 ^b^	1.6 ± 0.4 ^a^
150 °C	33 ± 6 ^b^	0.26 ± 0.01 ^c^	2.4 ± 0.3 ^b^

## Supplementary Materials

The following are available online at https://www.mdpi.com/2073-4360/13/3/398/s1, Figure S1: Elastic modulus (E′) during the ageing process of the doughs obtained after mixing (kept in closed containers) versus time.
