# Supplementary material for: Effects of Mould Temperature on Rice Bran-Based Bioplastics Obtained by Injection Moulding
Source: Polymers (Basel). 2021 Jan 27;13(3):398. doi: 10.3390/polym13030398 (PMC7866207; doi:10.3390/polym13030398)
Supplement: Supplementary file 1 [file polymers-13-00398-s001.pdf]

polymers
MDPI

Article

Effects of Mould Temperature on Rice Bran-Based Bioplastics Obtained by Injection Moulding

María Alonso-González [1,*], Manuel Felix [2], Antonio Guerrero [2] and Alberto Romero [1]

[1] Departamento de Ingeniería Química, Facultad de Química, Universidad de Sevilla, 41012 Sevilla, Spain; alromero@us.es
[2] Departamento de Ingeniería Química, Escuela Politécnica Superior, Universidad de Sevilla, 41011 Sevilla, Spain; mfelix@us.es (M.F.); aguerrero@us.es (A.G.)
* Correspondence: maralonso@us.es; Tel.: +34-635-313-411

Supplementary Materials

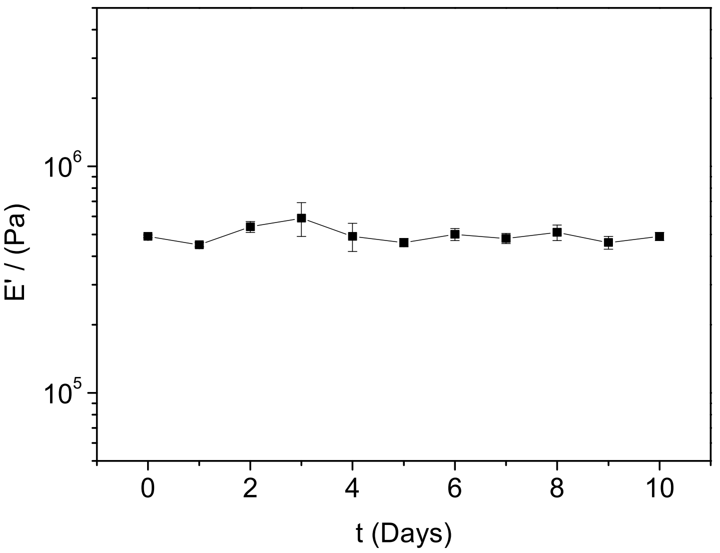

Figure S1. Elastic modulus (E') during the aging process of the doughs obtained after mixing (kept in closed containers) versus time. .

Citation: Alonso-González, M.; Felix, M.; Guerrero, A.; Romero, A. Effects of Mould Temperature on Rice Bran-Based Bioplastics Obtained by Injection Moulding. *Polymers* **2021**, *13*, 398. https://doi.org/10.3390/10.3390/polym13030398

Academic Editor: Alonso-González M.

Received: 13 January 2021
Accepted: 25 January 2021
Published: 27 January 2021

Publisher's Note: MDPI stays neutral with regard to jurisdictional claims in published maps and institutional affiliations.

Copyright: © 2021 by the authors. Licensee MDPI, Basel, Switzerland. This article is an open access article distributed under the terms and conditions of the Creative Commons Attribution (CC BY) license (http://creativecommons.org/licenses/by/4.0/).
